# Vitamin D Deficiency in Cystic Fibrosis

**DOI:** 10.1155/2010/218691

**Published:** 2010-01-28

**Authors:** William B. Hall, Amy A. Sparks, Robert M. Aris

**Affiliations:** Division of Pulmonary and Critical Care Medicine and the School of Medicine, The University of North Carolina at Chapel Hill, Chapel Hill, NC 27599, USA

## Abstract

Cystic Fibrosis is the most common inherited genetic respiratory disorder in the Western World. Hypovitaminosis D is almost universal in CF patients, likely due to a combination of inadequate absorption, impaired metabolism, and lack of sun exposure. Inadequate levels are associated with the high prevalence of bone disease or osteoporosis in CF patients, which is associated with increased morbidity including fractures, kyphosis, and worsening pulmonary status. Treatment goals include regular monitoring 25 hydroxyvitamin D (25OHD) levels with aggressive treatment for those with levels <75 nmol/L (<30 ng/mL). More research is needed to determine optimal supplementation goals and strategies.

## 1. Cystic Fibrosis

With an estimated incidence of 1 per 3000 live births cystic fibrosis (CF) is the most common inherited respiratory disease in the western world [1, 2]. CF is caused by dysfunction of the CF transmembrane conductance regulator (CFTR), a chloride channel present on epithelial cells. Thus, CFTR mutations affect the respiratory, gastrointestinal, hepatobiliary, and reproductive systems as well as sweat glands. Most patients with CF succumb to respiratory failure from chronic pulmonary infection. Environmental, nutritional, and socioeconomic factors as well as modifier genes may affect the clinical manifestations of the disorder. Although a single gene deletion, F508del, is responsible for a majority of the mutations causing CF worldwide, more than 1600 mutations in CFTR have been described [3]. While there is great geographic and ethnic variation in the frequency of the disorder, CF remains predominantly a disease in Caucasians. The mean survival age in CF patients continues to improve from 2 in the 1950s to 37 years currently with roughly half of all CF individuals being of adult age. Survival is closely associated with both pulmonary and nutritional status [4–7].

## 2. Diagnosis of Vitamin D Deficiency

Like with non-CF individuals, the 25 hydroxyvitamin D (25OHD) level determines the degree of vitamin D insufficiency and, following data published outside CF, a 25OHD level <75 nmol/L (30 ng/mL) is considered insufficient. All CF patients should have vitamin D levels checked annually, ideally by high-performance liquid chromatography (HPLC) mass spectrometry test. Serum concentrations of 1–25 dihydroxyvitamin D have little impact on the management of vitamin D problems in CF. These levels are often normal or elevated in the setting of vitamin D deficiency due to increased activity of renal 1-hydroxylase under the influence of elevated PTH [8]. As in the general population, serum 25OHD levels may display variability based on the time of year and latitude and should be checked in late fall or winter to determine the degree of deficiency. 

## 3. Magnitude and Causes of Vitamin D Deficiency in CF

CF represents the “perfect storm” for vitamin D deficiency. More than 20 studies have documented low levels of 25OHD from around the world and at many different latitudes. In recent studies from large CF Centers, >90% of patients have 25OHD levels < 75 nmol/L (30 ng/mL) [9, 10]. Table 1 summarizes many of the documented 25OHD levels over the last few decades. These data suggest a trend toward higher 25OHD levels over recent years (especially among larger studies), which likely reflects increased attention to 25OHD levels by most large CF centers. The continued presence of low 25OHD emphasizes the continued inadequate supplementation despite increased awareness.

**Figure 1:**
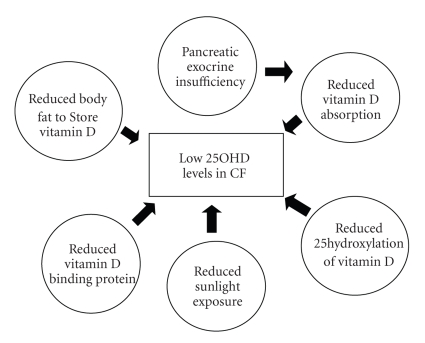
Causes of Vitamin D insufficiency in CF Patients [11].

**Figure 2:**
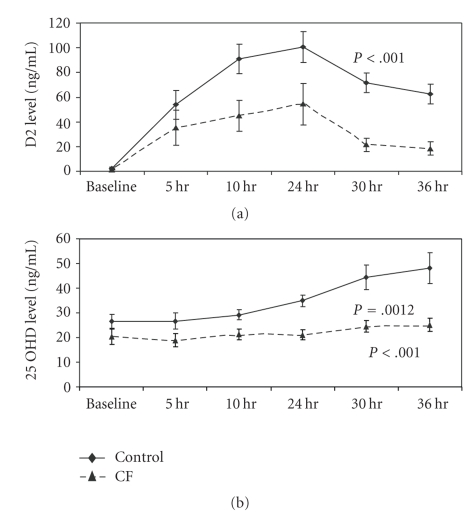
Impaired absorption of Vitamin D following a 2500 ug dose in in CF patients and controls [13].

The multifactorial etiology of low vitamin D levels in CF patients is illustrated in Figure 1. Despite routine oral supplementation, intake of vitamin D is often inadequate [10]. Ingested vitamin D is often not absorbed due to exocrine pancreatic insufficiency, present in 85% to 90% of individuals with CF [12]. Exocrine pancreatic insufficiency causes intestinal malabsorption of all fat soluble vitamins. Cystic fibrosis patients have impaired absorption of vitamin D from both plant sources (vitamin D2) and animal sources (vitamin D3) [8]. Figure 2 obtained from Lark et al. illustrates the decreased absorption from a single 2500 ug dose of vitamin D2 in CF patients despite pancreatic enzyme supplementation. Both decreased serum levels of vitamin D2 and 25OHD were noted [13]. With chronic supplementation, however, 25OHD levels do show some improvement with pancreatic enzyme and vitamin supplementation [14].

In addition to vitamin D malabsorption, CF patients exhibit impaired hepatic hydroxylation, which may affect metabolism of manufactured vitamin D and vitamin D absorbed through the gastrointestinal tract [15–25]. The kinetics of vitamin D metabolism have not been studied in CF in detail (i.e., tracer studies), but indirect data suggest that there may be accelerated excretion of vitamin D, possibly through enterohepatic dumping, before exposure to the hepatic 25-hydroxylase enzyme [13].

Decreased storage of both produced and consumed vitamin D may also be due to decreased levels of vitamin D binding protein (DBP) in CF patients, a phenomenon known to exist for 3 decades [26]. DBP shuttles vitamin D from the intestine to fat beds. The effects of low DBP on vitamin D levels and metabolism are not entirely clear. However, most 25OHD is carried by DBP and very little is free in serum. High concentrations of unbound DBP in normal patients may function as a reservoir for 25OHD [27].

It is likely that CF patients have decreased vitamin D synthesis. In the normal population, 90% to 95% of the vitamin D requirement comes from exposure to sunlight. Healthy individuals can obtain their vitamin D requirement by exposing either their hands, face, and arms, or arms and legs to sunlight. The amount needed is 2 or 3 times a week in the spring, summer, and fall to about 20% to 25% of the amount of sunlight it would take to cause a mild pinkness to the skin [8, 28]. Many CF patients actively avoid sunlight exposure due to photosensitivity from some antibiotics. Though normal individuals store vitamin D produced in the skin to be released during the winter, cystic fibrosis patients who are exposed to the sun may have little body fat and may store less vitamin D, further exacerbating the problem.

## 4. Manifestations of Vitamin D Deficiency in CF Patients

In children, severe vitamin D deficiency results in rickets, but its clinical presentation in CF is more subtle. Studies from non-CF populations show that vitamin D deficiency in adults causes secondary hyperparathyroidism, resulting in mobilization of mineral and matrix from the skeleton, and precipitating or causing osteomalacia, but this condition has rarely been described in CF [8]. Nonetheless, several studies have shown abnormally high PTH levels in CF, reduced numbers of osteoblasts on bone biopsies, and some evidence of prolonged mineralization lag times in some CF adults suggesting that mineralization defects, short of true rickets/osteomalacia, do occur [54].

The complete impact of low vitamin D levels in CF patients is yet to be determined. Though vitamin D likely has roles in muscle function, innate immunity, cardiovascular disease, diabetes, and some malignancies [55], there is no information on these outcomes in CF. In fact, vitamin D has been mainly studied in the context of CF bone disease. A recent meta-analysis by the University of Ottawa Evidence Based Practice Center (EPC) suggested a fair correlation between low vitamin D levels and Bone Mass Density (BMD) in healthy adolescents and adults, but such correlations have been hard to demonstrate in CF [56]. Inconsistent correlation may also be due to a myriad of nonvitamin D factors in CF that affect bone health as well as the known seasonal and lifetime variations in serum 25OHD levels.

## 5. Role of Vitamin D Deficiency in Bone Health in CF

While the connection between low 25OHD levels and low bone density in CF has been hard to verify, it seems highly likely that low vitamin D levels play a role in poor bone health that is very frequently seen in adults with CF. In 1979, 2 independent studies reported a decrease in bone mineral content in patients with CF as compared to age-matched controls [24, 25]. Dozens of additional reports of low bone mass and frank osteoporosis in patients with CF, particularly in adolescents and adults, have been written over the past 30 years [15, 16, 18, 43, 46–50, 57, 58]. The US CF Foundation 2007 Patient Registry of ~24,000 CF individuals reported a rate of bone disease in adults with CF of ~21% (along with 0.5% for bone fractures), a number which has increased by an order of magnitude in the last 7 years largely due to better screening and recognition of bone disease [1].

Despite better screening, large careful cross-sectional studies indicate that these rates almost certainly underestimate the true prevalence due to continued under-reporting. Studies from at least 7 countries have found that low BMD is common in both children and adults with CF, although adults tend to be more affected. In some studies, up to 69% of patients have been reported to have low bone mass, with 57% displaying bone density greater than 2 standard deviations below the mean for age-matched controls [15, 16, 20, 46, 49, 50, 57, 58].

Bone mineral accrual in CF is likely inadequate [18, 54, 59], especially from late childhood through young adulthood, when measured volumetrically by quantitative computed tomography (QCT) [59] or when corrected for bone volume (bone mineral apparent density (BMAD)). Here in particular, there is a concern that inadequate vitamin D contributes to poor bone health in CF.

Markers of bone formation and breakdown have improved the understanding of CF bone disease, which include the osteoblast markers bone-specific alkaline phosphatase and osteocalcin in addition to the osteoclast markers pyridinoline crosslinks and collagen N-telopeptides. Though these markers display significant variability with age, puberty, season, time of day, menstrual cycle, and amount of lung infection, they support the histomorphometric data demonstrating both increased bone breakdown and inadequate formation [16, 21, 42, 43, 60–63]. These data, when taken as a whole, then suggest that low BMD in CF is a result of inadequate bone formation and increased bone resorption, with low vitamin D as a likely contributor.

Both the degree of bone disease and degree of vitamin D deficiency appear to increase with age and severity of lung disease. A recent study showed a correlation between 25OHD levels and decreased FEV1 (forced expiratory volume in 1 second, an important measure of lung function) and nutritional status [64]. Several studies have demonstrated positive correlation of BMD with FEV_1_ [15, 43, 47, 48, 59]. Increases in fracture rates may occur as early as the late teens and early twenties, decades before increases are seen in the general population. While increased fractures are not solely due to vitamin D deficiency, this problem does contribute. Similar to the general population, fractures appear to occur earlier in CF women [48]. While fractures tend to affect the axillary skeleton to a greater extent than the appendicular skeleton, no site is unaffected. Resultant chest wall deformities and “splinting” due to pain from thoracic vertebral and rib fractures can inhibit effective cough and airway clearance. Ultimately, this may accelerate the decline in lung function in patients with CF.

Most end-stage CF patients get referred for lung transplantation in developed countries. Few data are available regarding the impact of lung transplantation on 25OHD. One study reported that 25OHD levels went up slowly after transplant suggesting that lung disease and systemic ramifications of chronic inflammation affect the absorption of precursor molecule or the 25-hydroxylase enzyme [42]. Unfortunately most patients already suffer from severe osteoporosis before transplant [19, 65–67]. Post lung transplant patients carry additional fracture risk [19, 65], which may be attributed to increased lung function and activity level. Transplant recipients appear to develop high-turnover osteoporosis, which appears to be linked to immunosuppressants rather than vitamin D insufficiency. This causes decreases in spine and femur BMD as high as 10% in posttransplant CF patients [19, 35, 66, 68, 69] and pathologic fracture rates after transplant as high as 37% to 42% [66, 70]. Regardless of the etiology, maximizing vitamin D status in the years before transplant will likely be key to improving bone health post-transplant.

## 6. Treatment of Vitamin D Deficiency

It is recommended that all CF patients target an optimum 25OHD level in the range of 75–150 nmol/L (30–60 ng/mL), but levels up to 250 nmol/L (100 ng/mL) are probably still safe. These targets are based on data outside of CF that demonstrate that parathyroid hormone (PTH) levels (a sensitive marker of serum ionized calcium levels) start to rise when 25OHD levels fall below 75 nmol/L (30 ng/mL) [69].

Trials have further reinforced the need to update vitamin D supplementation algorithms. A summary of all vitamin D related intervention research studies in the CF population is shown in Table 2. Most early trials failed to significantly change participants' serum 25OHD levels. Stephenson et al. were able to achieve 25OHD levels ≥ 50 nmol/l (20 ng/mL) in 92% of patients treated with, on average, 1800 IU D3; however only 18% of these participants reached the ideal 75 nmol/L (30 ng/mL) level [35]. Gronowitz et al. had encouraging results with the use of UVB lamps combined with D3 supplementation, raising the mean serum 25OHD level in this group of patients from 55 nmol/L (22 ng/mL) to 125 nmol/L (50 ng/mL) in 12 weeks [72]. However a recent trial by Khazai et al. saw only that 55% of patients reach repletion levels using UVB due to difficulties with compliance. Khazai et al. also showed a promising method in which 100% of participants treated with 50,000 IU D3 weekly for 3 months reached the target serum 25OHD level of >75 nmol/L (30 ng/mL) [29]. They also demonstrated some success with 50,000 IU D2, but to a lesser extent. Due to the limited sample size and short duration of this study, further research is needed in order to determine if this strategy will be beneficial for most CF patients.

The Khazai study illustrates the important point that vitamin D2 and D3 supplementation may not be equivalent. Non-CF patients receiving equipotent doses of D2 and D3 had similar initial 25OHD levels, though the levels declined more rapidly in those receiving D2 [73]. The superiority of D3 to maintain adequate 25OHD levels has also been suggested in earlier literature although this remains controversial [73–76].

**Figure 3:**
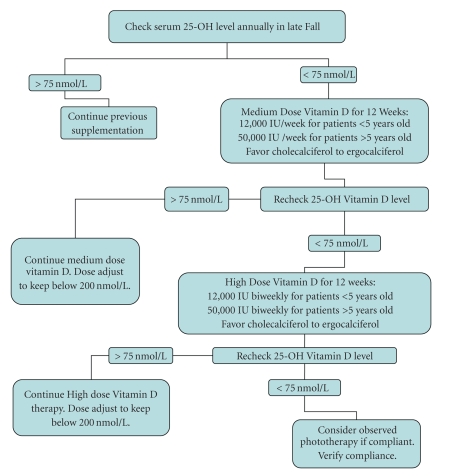
Suggested Vitamin D supplementation algorithm.

No superior supplementation strategy has been adequately validated by randomized controlled trials; so current strategies are based on expert review of available data. We suggest a modified and updated version of a previously published algorithm recommended by a recent consensus conference on CF-related bone health [77], which is outlined in Figure 3. Children younger than 1 year of age with low 25OHD levels should receive 8000 IU vitamin D per week; individuals older than 1 year should routinely receive 800 IU or more vitamin D per day. If circulating 25OHD concentrations are still <75 nmol/L (30 ng/mL) and patients are considered adherent to treatment, patients 5 years or older may be given the Medium Dose Regimen, which we define as 50,000 IU vitamin D once per week for 12 weeks. Patients younger than 5 years should be given 12,000 IU vitamin D once per week for 12 weeks. It is critical to recheck the 25OHD level while on supplemental therapy and not to allow a lapse in the repletion effort before the 25OHD level is rechecked. All treatments requires close follow-up to ensure that the 25OHD levels respond. Seasonal variation may need to be accounted for in the long-term repletion effort and considerable interindividual variation in the amount needed to achieve the goal level should be expected. Patients not responding to the Medium Dose Regimen, after adherence has been confirmed, should be given the High Dose Regimen, defined as 50,000 IU (or 12 000 IU for patients younger than 5 years) vitamin D biweekly and reassessment done after 12 weeks. The evolving data suggest that vitamin D amounts in the range of 3000–5000 IU/day may be needed for most adults with CF and this amount may be dosed daily or weekly. We currently favor vitamin D3 supplementation over vitamin D2 until further data are available.

Some patients will have inadequate levels despite these efforts. Increased exposure to sunlight or phototherapy without sun-blocking lotions may also be considered. Sunlight exposure should be short enough to prevent sunburn. Hands, face, and arms, or arms and legs should be exposed 2 or 3 times a week in the spring, summer, and fall to an amount of sunlight that is equivalent to about 20% to 25% of the amount it would take to cause a mild pinkness to the skin. Phototherapy with artificial ultraviolet lights such as a tanning bed or portable tanning units may be used, provided that the phototherapy unit has the component of UV-B that is responsible for making vitamin D in the skin. The manufacturer's guidelines for exposure dependent on skin type should be followed. It should be noted that adherence to this regimen is critical, as poor compliance has been implicated as a culprit in most negative studies.

## 7. Future Directions in Research

Despite our increasing knowledge about vitamin D, basic understanding regarding the association between vitamin D and BMD or fracture risk is still not completely clear. Studies are needed to determine the amount of orally administered vitamin D needed to maintain circulating concentrations of 25OHD above the target level of >75 nmol/L (30 ng/mL), but optimal dosing for clinically useful endpoints (e.g., fracture prevention and improving/stabilizing BMD) is needed. In all likelihood, caregivers will have to individualize doses to some extent to meet the needs of all of their patients. More research is needed to determine the short- and long-term consequences of vitamin D insufficiency in CF. The role of vitamin D repletion in inflammation in CF needs to be explored. Vitamin D ligands (VDLs) are upregulated in inflammatory disease states in various cell lines, and vitamin D's anti-inflammatory role has been documented in many diseases such as multiple sclerosis, rheumatoid arthritis, type I and II diabetes mellitus, lupus, psoriasis, and prostate cancer. While it is expected that findings from general populations with regard to low vitamin D status ramify to CF individuals, there is also concern that the anti-inflammatory activity of vitamin D levels may be even more important in CF vis a vis chronic lung infection and the systemic response that results. Phototherapy studies are also needed to better define the amount of exposure to UV-B radiation that is needed to maintain 25OHD levels > 75 nmol/L (30 ng/mL) without causing toxicity. 

## 8. Conclusions

CF patients have a particularly difficult time maintaining adequate vitamin D levels for a host of environmental, genetic, and circumstantial reasons. Added to this is a predisposition to osteoporosis compounding the impact of low vitamin D levels on morbidity and mortality. Physicians caring for CF patients should be proactive in monitoring 25OHD levels with aggressive treatment of those with low levels in an effort to prevent adverse long-term consequences. Consultants in endocrinology will be very helpful to CF pulmonologists in the management of difficult to treat CF patients as their knowledge of vitamin D metabolism is considerable. Current guidelines for repletion are similar to those in the general population (25OHD levels > 75 nmol/L [30 ng/mL]).

## Figures and Tables

**Table 1:** 25OH vitamin D levels in CF patients.

Ref	Year	Design	No. of pts	Mean Age	Mean/MEDIAN 25OHD Level
Khazai et al. [29]	2009	RCT	30	32.3	56.8 nmol/l
Fewtrell et al. [30]	2008	observational	32	9.8	63.8
Speeckaert et al. [31]	2008	observational	116	15.1	70.9
Hillman et al. [32]	2008	RCT	15	9.1 +/− 2.3	88.75
Wolfenden et al. [33]	2008	Retrospective Cohort	185	29 ± 9	58.8
Rovner et al. [10]	2007	Prospective case/control	101	14.8 + 4.2	51.7
Gordon et al. [34]	2007	Cross sectional	64	31.4 +/− 9.1	48.4
Stephenson et al. [35]	2007	Retrospective cohort	360	28 +/− 9	47
Boyle et al. [9]	2005	Prospective tx study	134	29.6 +/− 8.9	53.7
Chavasse et al. [36]	2004	Retrospective cohort	290	9 (no SD)	65
Haworth et al. [37]	2004	RCT	30	27.7	57.5
Brown et al. [38]	2003	Case control	10	25.6 +/− 6.5	58.6
Leifke et al. [39]	2003	Cross sectional	40	27.4 +/− 5.4	39.5
Gronowitz et al. [40]	2003	Observational	70	18.8	70.2
Flohr et al. [41]	2002	Observational	75	25.3	69.3
Aris et al. [42]	2002	Case control	50	28.3 +/− 7.8	52.4
Lark et al. [13]	2001	Case control	10	28.9 +/− 8.1	49.4
Elkin et al. [43]	2001	Observational	107	28 +/− 8	38
Grey et al. [44]	2000	Cross sectional	40	10.5 +/− 3.9	74.4
Mortensen et al. [45]	2000	Case control	11	9	55
Conway et al. [46]	2000	Observational	114	24.5	44.9
Haworth et al. [47]	1999	Observational	151	25.3 +/− 7.1	47.4
Aris et al. [48]	1998	Retrospective cohort	70	28.1	52.7
Henderson and Madsen [15]	1996	Observational	54	11.0	64.9
Bhudhikanok et al. [49]	1996	Case control	49	20.6	53% were <44.9
Rochat et al. [16]	1994	Observational	12	23.3	54.9
Grey et al. [50]	1993	Observational	16	23	44.9
Stead et al. [51, 52]	1988	Observational	31	24.5	25.0
Hanly et al. [53]	1985	Observational	20	18	21.3

**Table 2:** Vitamin D Therapy Trials in CF.

Article	Design	Patients	Intervention	Outcome	Limitations
Hillman et al. [32]	Double-blinded RCT	15 children aged 7–13	2000 D3 + 1 g Ca versus 2000 D3 versus 400 D3 + 1 g Ca versus 400 D3 for 6 months	No change in 25OHD, 1,25OHD levels in any arm	Short duration of treatment, small study, young patients only

Boyle et al. [9]	Open label CT	66 adult vitamin D deficient (25OHD ≤ 75 nmol/L [30 ng/mL]) patients	Sequential efforts of 50,000 IU D2 weekly for 8 weeks or twice weekly for 8 weeks	Only 5 (8%) patients achieved repletion (25OHD ≥ 75 nmol/L [30 ng/mL])	Many follow up 25OHD levels may have been drawn after supplementation ended.

Haworth et al. [37]	Double-blinded RCT	30 adult patients with BMD z score ≤ 1 in lumbar spine, femur or distal forearm	1 g cal + 1700 IU D3 (treatment) versus 900 IU D3 (control) over 12 months	Treatment group showed reduced rate of bone loss, no significant change in 25OHD level	Small sample size, changes in BMD not statistically significant

Brown et al. [38]	Open Label	10 adult CF patients10 healthy patients	5 microg calcitriol bid for 14 days	Increased short term study showing improved calcium absorption in both healthy and CF patients. In CF, calcitriol also decreased urine NTX in CF patients.	Short duration of treatment, Long term effects unknown. Calcitriol requires close monitoring to prevent hypecalciuria & hypercalcemia

Stephenson et al. [35]	Open Label, dose escalating study	215 CF adults patients with 25OHD ≤ 50 nmol/L (20 ng/mL)	400 IU D3, 800 IU D3, 1000 IU D3, >1000 IU D3, compliance counseling	92% achieved 25OHD ≥ 50 nmol/L (20 ng/mL) with average dose of 1800 IU/day Only 18% of these patients achieved 25OHD ≥ 75 nmol/L	No control group, no differentiation in outcome between intervention arms

Khazai et al. [29]	Open label RCT	30 CF adult patients	50,000 IU D2 weekly	100% D3 achieved 25OHD ≥ 75 nmol/L (30 ng/mL)	Small sample size, limited compliance in sun lamp arm
			50,000 IU D3 weekly,	60% D2 achieved 25OHD ≥ 75 nmol/L (30 ng/mL)	
			2%–5% UVB lamps	55% sunlamps achieved 25OHD ≥ 75 nmol/L (30 ng/mL)	

Green 2008 [71]	Retrospective chart review	262 pediatric CF patients	50,000 IU D2 1X weekly, 2X weekly or 3X weekly for variable lengths of time	33% weekly achieved 25OHD ≥ 75 nmol/L (30 ng/mL) 26% twice weekly achieved 25OHD ≥ 75 nmol/L (30 ng/mL) 43% thrice weekly 25OHD ≥ 75 nmol/L (30 ng/mL)	While there was some improvement in levels, no treatment was significantly better than controls

Gronowitz et al. [72]	Open Label Controlled Trial	30 pts aged 9–40 mean initial 25OHD 53 nmol/L (21.2 ng/mL) in control and 55 nmol/L (22 ng/mL) in treatment at baseline	Routine Supplementation (400-2250 IU D3 daily) versus Routine Supplementation + UVB lamps for 12 weeks	25OHD level post 8 weeks: Control 48 nmol/L (19.2 ng/mL) Intervention 110 nmol/L (44 ng/mL) Post 12 weeks: Control 63 nmol/L (25.2 ng/ml) Intervention 125 nmol/L/mL (50 ng/mL)	Limited compliance to treatment regimen due to skin type side effects with prolonged UVB exposure
